# Selfie and the City: A World-Wide, Large, and Ecologically Valid Database Reveals a Two-Pronged Side Bias in Naïve Self-Portraits

**DOI:** 10.1371/journal.pone.0124999

**Published:** 2015-04-27

**Authors:** Nicola Bruno, Marco Bertamini, Federica Protti

**Affiliations:** 1 Dipartimento di Neuroscienze, Università di Parma, Parma, Italy; 2 School of Psychology, University of Liverpool, Liverpool, United Kingdom; 3 Università Suor Orsola Benincasa, Napoli, Italy; University of Lincoln, UNITED KINGDOM

## Abstract

Self-portraits are more likely to show the artist’s right than left cheek. This phenomenon may have a psychobiological basis: Self-portraitists often copy their subject from mirrors and, if they prefer to present their left cheek (more expressive due to right-lateralization of emotions) to the mirror, this would result in a right-cheek bias in the painting. We tested this hypothesis using SelfieCity (3200 selfies posted on Instagram from December 4 through 12, 2013 from New York, Sao Paulo, Berlin, Moskow, and Bangkok), which includes two selfie-taking styles: a “standard” (photograph of selfie-taker) and a “mirror” (photograph of mirror reflection of selfie-taker) style. We show that the first style reveals a left cheek bias, whereas the second reveals a right cheek bias. Thus side biases observed in a world-wide, large, and ecologically valid database of naïve self-portraits provide strong support for a role of psychobiological factors in the artistic composition of self-portraits.

## Introduction

According to surveys of art books and exhibitions, when composing self-portraits artists are more likely to paint their faces showing the right than the left cheek[1–2]. This bias can be quantified at about 55–65% for non-frontal self-portraits painted from the 15th to the 19th century, and is no longer present in the 20th century [3–4]. Although the causes of the bias remain controversial [5], it seems clear that mirrors are part of the explanation. Presumably, before the invention of photography self-portraitists stood in front of the canvas and used mirrors to see themselves, and these mirrors were more often placed on their left. Thus, artists more often presented their left cheek to the mirror, which resulted in a right-cheeked mirror image that they then copied. But why would artist choose this arrangement?

One possibility is that placing the mirror on the left is favored due to biomechanical constraints (i.e., it is more natural for a right-handed artist as it avoids occlusion of the subject from the hand holding the brush) or to culturally induced studio conventions. Alternatively, the bias may have a psychobiological basis: It may depend on biologically determined asymmetries in facial expressiveness [6]. Due to right-hemispheric dominance for the expression of emotions [7], artist may have a tendency to prefer studio arrangements that make it easy for them to showcase their more expressive side. Supporting a psychobiological basis, there is evidence for the opposite left cheek bias when artists paint portraits rather than self-portraits [8–10]. However, these findings cannot be considered definitive as there is rarely a way of knowing what techniques were used in a given portrait or self-portrait in earlier centuries.

In two recent papers [3] [11], we proposed that a useful testbed for hypotheses about psychological factors in portraiture can be found in databases of selfies, photographic self-portraits taken with a digital camera. Selfies are very simple and quick to produce, they are typically done by non-professional photographers, and are often taken for the purpose of posting on web-based social media. Painted self-portraits, conversely, require considerable skills, training, and time, and are made for obviously different purposes. If side-biases observed in painting can be found in selfies as well, this would represent strong evidence that they depend on psychobiological rather than on mechanical or cultural factors. One of our previous papers [3] documented a side bias in selfies taken in the laboratory by Italian participants with no training in photography. Although this finding is what one would expect if the bias had a psychobiological basis, whether it can be generalized to more realistic, ecologically valid, and culturally diverse conditions remains to be determined.

In the present paper, we present novel evidence from a dataset of 3200 selfies from five different major cities in Western and Eastern Europe, North and South America, and Asia. Crucially, this dataset features two, easily discernable, selfie-taking styles: a “standard” style whereby the selfie-taker pointed the digital camera towards himself or herself, and a “mirror” style whereby the taker stood in front of a mirror and photographed his or her mirror image. (Selfies can be taken using ordinary cellphones or smartphones. Users of smartphones may know that when taking a selfie using the front camera, the preview shows the mirror image of the taker. However, when saving the photograph the smartphone saves it as if taken by the back camera, such that in the saved selfies the left cheek corresponds to the actual left cheek of the taker. Thus, we can be sure that in standard selfies left cheeks in the picture correspond to left cheeks of takers, even if there is no way of knowing which device was used.) The standard and mirror styles are easily distinguished because in the latter the camera is visible in the selfie, whereas it is not in the former. This feature of the database, along with its demographics, makes it the ideal testbed for our psychobiological hypothesis. Independent of country of origin, standard-style selfies should show a left cheek bias, whereas mirror-style selfies should show the opposite, right cheek bias.

## Methods

We analyzed 3200 photographic self-portraits spontaneously uploaded by selfie-takers from five major cities (New York, Sao Paulo, Berlin, Moskow, and Bangkok) on the online photo-sharing social network Instagram from December 4 through 12, 2103. A CUNY research laboratory led by Lev Manovich selected the selfies for the purposes of the publicly available social-data visualization project SelfieCity. All uses of the selfies complied with Instagram’s privacy policy (http://instagram.com/about/legal/privacy/). The selection process employed a mixture of automatic and human scoring to obtain a total of 640 selfies from each city (for details see http://selfiecity.net and [12]). The SelfieCity database includes a statistical web application, called SelfieExploratory, which provides filters for visualizing selfies within predefined categories. For instance, after applying the relevant filter, one obtains counts of females (2070), males (1076), and of selfie-takers whose sex cannot be determined with certainty (54). Most importantly for the purposes of the present paper, applying the filter for head turn reveals that the database contains more selfies with the head turned to the left (1686) than to the right (1514), which is consistent with the presence of a side bias.

This overall difference however is difficult to interpret for two reasons. First, as mentioned in the introduction, a quick perusal of the photographs immediately reveals two selfie-taking styles, standard and mirror, which make opposite predictions concerning the side bias and should therefore be kept separate. Second, head turn was measured in degrees to the left or to the right as a continuous variable. We have been unable to locate specific information on the algorithm that was used to compute this measure, although we assume that it was based on comparisons of eye, nose, and mouth positions as computed from the face analysis software (Orbeus Inc.) that was used to detect other features of interest such as, for instance, the degree of head tilt, the presence of glasses, and so on. Although clever, this measure is of limited interest in terms of compositional choices, for small differences (say, of the order of 1 or 2 degrees) matter little when the head is clearly turned in one direction—suggesting a clear choice for a three-quarter pose to the left or right—but turn out to matter a lot more when the head is only barely turned in one direction. For instance, if a photograph is coded as turning 1 degree to the left, this will be then counted in the “left” category. However, this may as well result from choosing a frontal pose, plus random error in the measurement or imprecise placement of the face relative to the camera. To address these problems, we re-analyzed the whole database using the classification employed in our previous paper, which parsed selfies into five categories: unambiguously facing left or right, slightly facing left or right, or frontal (for details see [3]).

Using the website, we inspected all selfies individually and recorded the city of origin, the selfie-taker sex, the posing choice, and the selfie-taking style. City of origin was derived by setting the appropriate filter on the web application. Sex, posing choice, and selfie-taking were determined based on the appearance of the individual in the photograph and on whether the camera was visible or not. We excluded a total of 83 photographs for one or more of the following reasons: The photograph was not a selfie (for instance, we found a picture of an infant on an infant seat, and the picture of a cartoon character’s face on a man’s sweater), the photograph featured two individuals, the face was blurry or occluded, the sex was uncertain, or the selfie-taker had uploaded more than one selfie (in this case, we scored only the first encountered in the database).

## Results

Our reanalysis revealed that as many as 2425 selfies displayed a clear turn toward the left or the right (see Table 1). The prevalence of frontal selfies (less than 10%) is much smaller than the 23% we observed previously [3]. This suggests that in-lab selfie taking may be only partly representative of the real life behavior and reinforces our claim that this database provides a more ecologically valid instance. Given this result, to provide the most stringent test of our hypothesis we limited the analysis to the unambiguous poses.

**Table 1:** Counts of the five posing categories in our re-analysis of the SelfieCity database.

clear left	slight left	frontal	slight right	clear right
1213	237	221	244	1202

**Fig 1:**
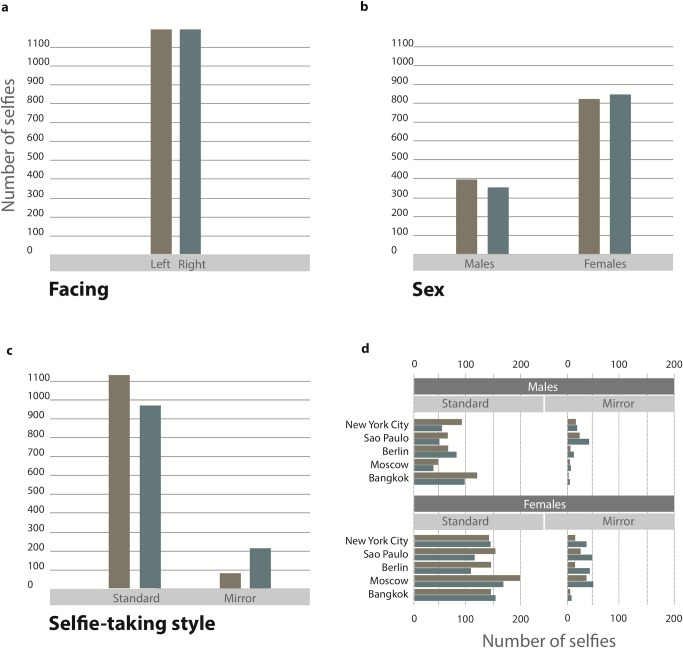
Results Frequencies of selfies showing the right (purple) or left (pale blue) cheek (a) in the SelfieCity database, as a function of selfie-taker sex (b), selfie-taking style (c), and city of origin (d). Only selfies that were coded as unambiguously showing one cheek more than the other were included in this analysis (about 2400 selfies, see text for details).

The results are displayed in Fig 1. No obvious posing biases emerge from comparing overall counts for left and right and as a function of taker sex (top row, 1a and 1b). However, a difference is revealed when dividing the data according to selfie-taking style. Standard selfies show a clear bias for showing the left cheek, whereas mirror selfies show the opposite right cheek bias (bottom row, 1c). The distribution of observed frequencies is extremely unlikely if pose and selfie-taking style were independent, chi-square(1) = 58.9, which corresponds to a probability of about once in ten thousand billions. The bias for the left cheek in standard selfies (about 53%) is smaller than the bias for the right cheek in mirror selfies (about 70%). Presumably, this is due to the mechanics of selfie-taking in the two styles. In standard selfies, holding the phone with the right hand makes it somewhat harder to present the left cheek to the camera, wheras in mirror selfies the phone is typically held more centrally near the body. The two-pronged bias is essentially the same for males and females in standard (about 54% vs about 53%) and mirror (about 70% vs about 66%) selfies. Finally, the two-pronged bias is essentially stable across the five cities (1d), with the only exceptions of Berlin males and Bangkok females for standard style selfies.

## Discussion

These results document a two-pronged side bias from a world-wide, large, and ecologically valid database of photographic self-portraits. Our re-analysis of the SelfieCity database, which includes 3200 selfies spontaneously uploaded on Instagram from five world major cities, revealed two, easily distinguishable selfie-taking styles corresponding to opposite predictions concerning the hypotesized side bias. Consistent with our hypothesis, we observed that standard-style selfies show a left-cheek bias, whereas mirror-style selfies show a right-cheek bias. The two biases are remarkably stable across different cities and between males and females, with inversions only for standard-style selfies for Berlin males and Bangkok females. These local inconsistencies may reflect cultural differences or, more likely, random variations within smaller subsets of the whole database. Overall, we interpret these results as providing remarkably convincing evidence for a, presumably unconscious, culture-independent preference for displaying one’s left cheek. Lateral asymmetries in processing faces have been observed in non-human species (for a review see [13]). That comparable asymmetries can be observed in a spontaneous, quasi-artistic behavior such as selfie-taking provides remarkably convincing evidence for a psychobiological account of compositional choices in self-portraits.
